# Modeling human neurodevelopmental diseases with brain organoids

**DOI:** 10.1186/s13619-021-00103-6

**Published:** 2022-01-04

**Authors:** Xiaoxiang Lu, Jiajie Yang, Yangfei Xiang

**Affiliations:** School of Life Science and Technology, ShanghaiTech University, Shanghai, 201210 China

**Keywords:** Brain organoids, Neurodevepmental diseases, Disease modeling, Stem cells, Gene editing

## Abstract

Studying the etiology of human neurodevelopmental diseases has long been a challenging task due to the brain’s complexity and its limited accessibility. Human pluripotent stem cells (hPSCs)-derived brain organoids are capable of recapitulating various features and functionalities of the human brain, allowing the investigation of intricate pathogenesis of developmental abnormalities. Over the past years, brain organoids have facilitated identifying disease-associated phenotypes and underlying mechanisms for human neurodevelopmental diseases. Integrating with more cutting-edge technologies, particularly gene editing, brain organoids further empower human disease modeling. Here, we review the latest progress in modeling human neurodevelopmental disorders with brain organoids.

## Background

The brain is the most complex organ in the human body. The human brain is substantially distinct from other species, including rodents and nonhuman primates, regarding brain size, shape, cell diversity, cognitive functions, and many other aspects (Rakic 2009). There are billions of highly specialized cells in the human brain that intertwine with each other to establish complex functions, yet the cellular diversities and their communications remain as largely unanswered questions that await extensive investigation (Sun and Hevner 2014). The complexity of the human brain, technical challenges to study it, and ethical issues involved, make the human brain one of the most mysterious organs to decipher (Bystron et al. 2008).

Abnormal differentiation and maturation of brain cells cause neurodevelopmental diseases. Autism spectrum disorder (ASD), schizophrenia (SZ), and microcephaly are some of the typical neurological diseases that show intellectual impairments, mental problems, and disabilities. These diseases may also display many other symptoms, including organ defects, different facial features, and sometimes lethality. Overall, neurodevelopmental diseases exert a tremendous impact on patients’ personal lives and society as a whole (The Lancet 2017; Vigo et al. 2016). Animal models have been serving as a critical platform for understanding various neurodevelopmental diseases. Nevertheless, fundamental differences between human and model organisms also call for the establishment of human models to investigate human disorders (2015; Zhao and Bhattacharyya 2018).

One critical step in the journey of human brain modeling was the successful conversion of human embryonic stem cells (hESCs) into neural precursors and mature neurons in monolayer cultures (Reubinoff et al. 2001; Zhang et al. 2001). While two-dimensional (2D) neural cultures provide important platforms for investigating the human nervous system, they still fall short in several aspects: 1) 2D cultures may produce relatively more homogenous cell populations, thus lacking the feature of complex cellular diversity in a real brain; 2) the absence of spatial organizations of diverse cell types may affect cell-cell communications and lead to inappropriate microenvironments for development; 3) given the absence of cellular diversity and their spatial organization, the establishment of complex and functional neural circuits in 2D culture is hardly practical.

Largely benefited from the progression in stem cell and developmental biology, the establishment of human brain organoids opens a new avenue to explore human neural development and diseases in a dish. Three-dimensional (3D) brain organoids derived from human pluripotent stem cells (hPSCs) can reconstruct the complex cellular composition, their spatial organization, and neural functions in the human brain (Lancaster et al. 2013). Over the past years, brain organoids have been widely applied to study normal and abnormal developmental processes of the human brain. Here, we review the advances in human brain organoid technology and their applications in deciphering human neurodevelopmental diseases. We also discuss current technical limitations and potential efforts required for addressing the challenges.

## Brain organoids: emergence and progress

The intrinsic capability of stem cell progenies to self-organize enables the production of 3D organoid models recapitulating various organs or tissues in the body. Organoids contain not only cellular diversities relevant to the in vivo counterparts, but importantly, their spatial organizations as well as corresponding physiological functions (Eiraku et al. 2011; Lancaster et al. 2013; Nakano et al. 2012; Ootani et al. 2009; Spence et al. 2011; Takasato et al. 2015). Brain organoids begin with the formation of embryoid bodies (EBs). Under adherent culture conditions, EBs could generate neural rosettes, a typical type of cell organization composed of polarized neural progenitor cells (NPCs) (Zhang et al. 2001). This early observation nicely implicated the potential of human neural cells to self-organize during differentiation, a fundamental prerequisite for brain organoid development in 3D culture.

**Fig. 1:**
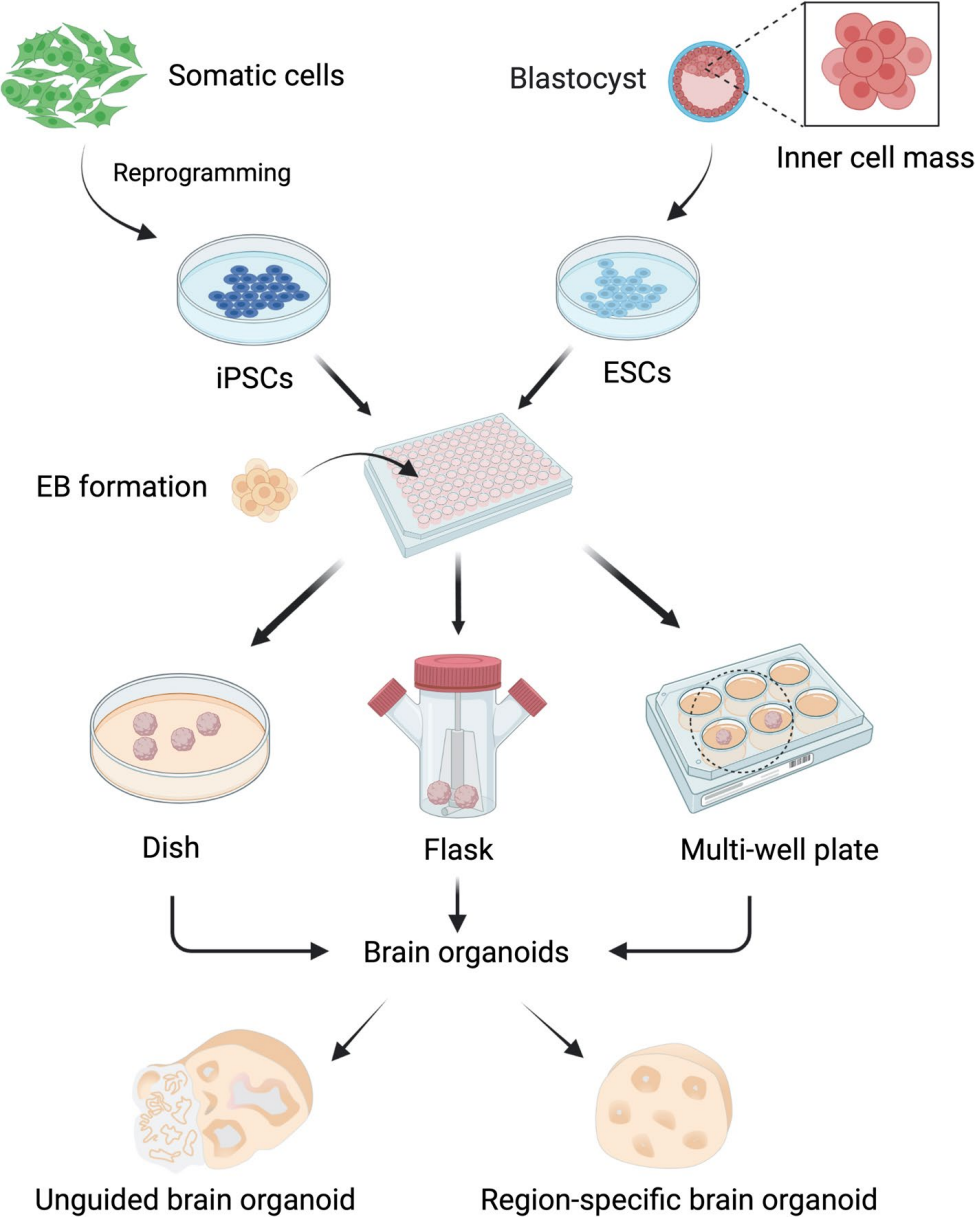
Methods for generating human brain organoids. Induced pluripotent stem cells (iPSCs) reprogrammed from somatic cells and embryonic stem cells (ESCs) derived from blastocytes both are pluripotent stem cells (PSCs). PSCs are dissociated into single cells to form floating embryonic bodies (EBs). EBs are then transferred to petri dish, spinning flask, low-attachment multi-well plate, or other bioreactors for further culture. Alternatively, PSCs can be detached as colony clumps to form EBs. EBs develop into brain organoids with or without the assistance of extracellular matrix (e.g., Matrigel), under the conditions of spinning or static culture. Eventually, PSCs can develop into whole-brain organoid by spontaneous differentiation or brain-region-specific organoids through guided differentiation

Current methods for brain organoids generation can be classified into two categories (Kelava and Lancaster 2016; Pașca 2018; Qian et al. 2019; Xiang et al. 2021): 1) Unguided brain organoids with heterogeneous brain region identities (Lancaster et al. 2013), or 2) guided brain organoids patterned by defined neural induction factors (Eiraku et al. 2008; Kadoshima et al. 2013). Brain organoids can be cultured with or without the addition of extracellular matrix (e.g., Matrigel), in bioreactors or tissue culture dishes, and under spinning or static conditions (Lancaster et al. 2013; Paşca et al. 2015; Qian et al. 2016; Xiang et al. 2017) (Fig. 1). Further modifications of brain organoid cultures have emerged as well, e.g., air-liquid interface techniques have been applied to enhance gas and nutrient exchange, thus assisting better structural and functional development and maturation of brain organoids (Giandomenico et al. 2019; Neal et al. 2018).

### Cerebral organoids and region-specific brain organoids

Cerebral organoids are among the most commonly applied types of brain organoids, which are generated from the unguided neural induction approach and contain independent and discrete brain region-like tissues (i.e., not region-specific). Albeit the fact that cerebral organoids may show higher heterogeneity compared to region-specific brain organoids, they mainly display structural and functional similarities with the human developing cerebral cortex, with well-organized apical-basal polarity, neuronal migration, and functional maturation in the 3D organoid tissue (Lancaster et al. 2013). An extensive survey of single-cell transcriptome from cerebral organoids has shown a broad diversity of cells relevant to endogenous counterparts can be produced and mature (Quadrato et al. 2017).

Region-specific brain organoids, also referred to as brain-region-specific organoids, are generated from various guided differentiation approaches. Multiple types of region-specific brain organoid systems have been established, including those specifically recapitulating the development of the human cerebral cortex, ventral telencephalon, optic cup, thalamus, hypothalamus, midbrain, choroid plexus, striatum, etc. (Benito-Kwiecinski et al. 2021; Huang et al. 2021; Kadoshima et al. 2013; Miura et al. 2020; Pollen et al. 2019; Qian et al. 2016; Qian et al. 2020; Xiang et al. 2021). These region-specific brain organoids differ from each other in terms of distinct cellular compositions and their unique physiological functions. For instance, an organoid model resembling the choroid plexus, the most dorsal region of the telencephalon, has recently been established, which shows properties of blood-cerebrospinal fluid (CSF) barrier and CSF-like fluid secretion (Pellegrini et al. 2020).

### Advancing the brain organoid technologies

Brain organoid technologies still face multiple challenges, among which the lack of vascularization is one of the critical issues affecting the long-term maturation of brain organoids. One approach to address this limitation is organoid transplantation. When transplanting cerebral organoids into the adult mouse brain, functional blood vessels and neuronal networks can be established in the grafts (Mansour et al. 2018). Functional vascularization is vital for the growth of brain organoid grafts (Cakir et al. 2019; Mansour et al. 2018; Shi et al. 2020). In addition, through introducing hESCs that ectopically express ETV2 into human cortical organoids (Cakir et al. 2019), coculturing cerebral organoids with induced pluripotent stem cells (iPSCs)-derived endothelial cells (ECs) or with human umbilical vein endothelial cells (HUVECs) (Pham et al. 2018; Shi et al. 2020), vasculature-like structures can be reconstructed in human brain organoids.

More complex brain organoid systems have been developed recently. By fusing/assembling distinct region-specific brain organoids, various cross-talk between human brain regions can be modeled in a dish. These particular systems, also referred to as assembloids, are capable of recapitulating directed cell migration and axonal projection in the developing human brain, including tangential migration of human interneurons from ventral forebrain to dorsal forebrain (Bagley et al. 2017; Birey et al. 2017; Xiang et al. 2017), reciprocal projections between cerebral cortex and thalamus (Xiang et al. 2019), cortico-striatal projections (Miura et al. 2020), and cortico-spinal projections to control muscle contraction (Andersen et al. 2020). It is conceivable that these complex brain organoid systems will offer more sophisticated platforms to decipher human brain function and dysfunction.

## Brain organoids: models of neurodevelopmental disease

The emergence of organoid technologies has provided an opportunity to closely investigate the pathogenesis of human neurodevelopmental diseases in a dish at molecular, cellular, and functional levels, particularly for diseases that may manifest differential phenotypes between human and animal models. Indeed, over the past decade, brain organoids have been widely applied to understanding brain developmental diseases, especially in combination with other cutting-edge technologies like gene editing, genome-wide screening, imaging, etc. (Etournay et al. 2016; Freedman et al. 2015; Okkelman et al. 2017; Ringel et al. 2020). In the following sections, we discuss how brain organoids can facilitate understanding neurodevelopmental diseases, including microcephaly-related disease (e.g., autosomal recessive primary microcephaly, MCPH), autism spectrum disorder (ASD), Rett syndrome (RTT), Timothy syndrome (TS), tuberous sclerosis complex (TSC), and Down syndrome (DS). These studies demonstrate that brain organoids can serve as an important platform to unravel phenotype, etiology, and even potential therapy for human neurodevelopmental diseases.

### Autosomal recessive primary microcephaly (MCPH)

MCPH is a neurodevelopmental disease in which head circumference is markedly reduced, directly characterized by a smaller cerebral cortex (Hanzlik and Gigante 2017). Extensive studies have demonstrated that numerous causative factors can lead to microcephaly, including genetic mutations (e.g., ASPM, CDK5RAP2, IER3IP1, Asparaginyl -tRNA synthetase1 (NARS1), KNL1, etc.), and prenatal exposure to toxins or pathogens (e.g., Zika virus) (Jayaraman et al. 2018). Model organisms like mice have been used to examine microcephaly-related pathogenesis. However, it is difficult to establish a realistic animal model for microcephaly with mice as mutant mice failed to recapitulate severely reduced brain size as seen in human patients (Barrera et al. 2010; Lui et al. 2011; Mahmood et al. 2011; Pulvers et al. 2010).

To model MCPH, iPSCs of a severely affected microcephaly patient (Lancaster et al. 2013) were generated. Patient-derived cells were confirmed to carry heterozygous truncating mutations of the CDK5RAP2 gene. Compared to wild-type cerebral organoids, CDK5RAP2-mutant cerebral organoids showed premature neural differentiation at the expense of progenitor pools, thus leading to smaller sizes of brain organoids. This microcephaly-like phenotype was unlikely an artificial effect of cell line discrepancies, as the reduced organoid size could be rescued by CDK5RAP2 overexpression, and could be reproduced by RNAi-mediated knockdown of CDK5RAP2 (Lancaster et al. 2013). iPSCs-derived cerebral organoids with mutations in genes KNL1 and NARS1 also displayed a reduction in neural progenitors and defect in the formation of neural rosette structure, which might be relevant to microcephaly (Omer Javed et al. 2018; Wang et al. 2020). Besides impairing neural progenitors, it was found NARS1 mutation may also cause a delay in neurodevelopment (Wang et al. 2020). iPSCs with biallelic ASPM mutations were used to produce human cortex-like brain organoids, which were cultured for up to 3 months. ASPM-mutant brain organoids displayed less-organized neuroepithelium structures, fewer ventral radial glial (vRG) cells, outer radial glial (oRG) cells, and defective layer lamination compared to wild-type brain organoids (Li et al. 2017a). This study is in line with a previous observation that mutations in Drosophila ASPM orthologue, Asp, might experience a deficiency in spindle orientation and interkinetic nuclear migration (INM) of neural progenitor cells (Rujano et al. 2013). Functionally, at later developmental stages, ASPM mutation also led to less synchronized neuronal activities in brain organoids (Li et al. 2017a).

Ablation in the WDR62 gene, the second most common causative gene for MCPH that encodes a centrosomal protein, resulted in premature differentiation of NPCs and a reduction in oRG proliferation. Importantly, the WDR62-CEP170-KIF2A pathway was identified as a critical contributor to microcephaly basing on studies in cerebral organoids and mice (Zhang et al. 2019). Through a brain organoid-based screening, it was found that IER3IP1, encoding a protein localized to the endoplasmic reticulum (ER), was a crucial regulator for brain growth (Esk et al. 2020). IER3IP1-knockout cerebral organoids showed a decrease in sizes of neural rosettes and organoids; genes associated with the ER-associated protein degradation (ERAD) pathway were selectively up-regulated in IER3IP1-knockout cerebral organoids as compared to wild-type controls (Esk et al. 2020). Overall, brain organoids have been well applied to model MCPH-related phenotypes.

Human brain organoids have also been applied to understand pathogen-caused microcephaly. Multiple lines of investigations have revealed that when exposed to Zika viruses, brain organoids will exhibit severe defects in the proliferation of neural progenitors, leading to abnormal neurogenesis and cell death (Cugola et al. 2016; Garcez et al. 2016; Qian et al. 2017; Qian et al. 2016). Using human forebrain organoid it was found that ZIKA virus-encoded protein NS2A reduced radial glial cell proliferation by causing deficits in adherens junctions and scaffolding of radial glial fibers (Yoon et al. 2017). Another two proteins NS4A and NS4B that suppress the Akt-mTOR pathway were verified to cause defective neurogenesis and aberrant activation of autophagy in human fetal neural stem cells (Liang et al. 2016). Together, these studies demonstrate that deep insights into MCPH, caused by either genetic mutations or pathogen infections, can be readily gained through the lens of human brain organoids.

### Autism spectrum disorder (ASD)

ASD is a heterogeneous brain disorder, caused by a considerable pool of genetic mutations (Hallmayer et al. 2011). ASD patients exhibit repetitive behaviors and impaired social interaction. Emerging evidence suggests that many of the ASD-associated genes are involved in cellular functions at different developmental stages and in different cell types of the embryonic brain (Basu et al. 2009; Iossifov et al. 2014).

To establish human models of ASD, telencephalic organoids were generated from iPSCs derived from patients and familial controls (Mariani et al. 2015). Interestingly, transcriptome analyses of ASD patient-specific organoids revealed a significant decrease in cell cycle length and an increase in the number of inhibitory synapses compared to the control. More progenitors and neurons of the GABAergic lineage were observed in ASD patient-specific organoids. Importantly, FOXG1, a transcription factor critical for telencephalon development and associated with atypical Rett syndrome and small brain size (Ariani et al. 2008; Kortum et al. 2011), was found abnormally up-regulated in ASD patient-specific organoids, and lentiviral-mediated knockdown of FOXG1 was able to rescue the aberrant high production of GABAergic neurons in ASD patient-specific organoids (Mariani et al. 2015).

A recent study by directly inducing ASD iPSCs into neurons and forebrain organoids revealed a temporal dysregulation of specific gene networks that caused marked abnormalities of cortical neuron development (Schafer et al. 2019). Maturing ASD neurons showed aberrant growth dynamics and more nerve branches. Importantly, skipping the NSC stage by direct iPSC-to-neuron conversion can avoid ASD-associated phenotypes in neurons (Schafer et al. 2019). CDH8, a gene encoding for a chromatin-remodeling factor, was also a causative gene for ASD (Wang et al. 2017). Cerebral organoids from heterozygous knockouts (CHD8+/−) iPSCs and controls (CHD8+/+) were established and compared. The study suggested that CHD8 mutation altered the expression of DLX genes, key regulators in GABAergic interneuron development. Pathway analysis of differentially expressed genes also revealed dysregulation in WNT/β-catenin signaling (Wang et al. 2017).

Utilizing brain organoids, it has been found that alterations in various genes and pathways are associated with ASD, among which a shift in GABAergic lineage determination was commonly observed in different systems (Marchetto et al. 2017; Mariani et al. 2015; Russo et al. 2018; Wang et al. 2017). Besides neurons, other non-neuronal contributors of ASD, like astrocytes and microglia, have also gained attention (Petrelli et al. 2016; Tan et al. 2020; Wang et al. 2021). Modeling such aspects with brain organoids, however, still remain challenging. Astrocytes in brain organoids mature only after long-term culture (Paşca et al. 2015; Sloan et al. 2017), and the presence of microglia in brain organoids is sparse, if any, and is hardly controllable (Ormel et al. 2018). Thus, optimized differentiation approaches or co-culture settings are to be developed to incorporate more cellular components for the purpose of a more complete ASD modeling.

### Rett syndrome (RTT)

RTT is a severe neurodevelopmental disorder, which is almost exclusively found in females and characterized by mental retardation and aberrant behavior. The primary cause for RTT is mutation in the X-linked gene methyl-CpG-binding protein 2 (MECP2) (Amir et al. 1999; Chahrour and Zoghbi 2007). MECP2 gene is abundantly expressed in brain neurons and is related to neuronal morphology and functional maturation (Jung et al. 2003).

In the past decades, post-mortem human brain samples (Colantuoni et al. 2001) and transgenic mouse models (Chen et al. 2001) have been used to study RTT. However, limited human brain samples and partially-presented phenotypes in animal models pose challenges for a full understanding of RTT. To better model human-specific phenotypes, neural cells were prepared from patient-derived iPSCs or MECP2-edited hESCs as monolayer cultures. These in vitro RTT models showed impaired human neural maturation, including fewer synapses, smaller soma size, and functional defects (Ananiev et al. 2011; Kim et al. 2011; Li et al. 2013; Marchetto et al. 2010; Tang et al. 2016; Xiang et al. 2020).

Brain organoids further assist to understand RTT etiology in a 3D developmental context. RTT patient iPSCs-derived cerebral organoids explained how the deficiency in MECP2 function affects neural progenitors and neuronal migration through regulation of miR-199 or miR-214 (Mellios et al. 2018). Human medial ganglionic eminence (MGE) organoids (hMGEOs) and human cortical organoids (hCOs) generated from MECP2-wild type and MECP2-mutant hESCs revealed a complex region-specific and cell-type-specific transcriptional dysregulation by MECP2 mutation (Xiang et al. 2020).

Along with the development of various region-specific brain organoids, fused brain organoids/assembloids have been applied to understand developmental processes, including those in a pathological background. For instance, dorsal, ventral, and assembled forebrain organoids were derived from RTT patient-derived iPSCs carrying R255X mutation (Gomes et al. 2020). Premature development of the deep-cortical subplate was observed, yielding neurons with functional deficits. This study also suggests a negative impact of MECP2 mutation on tangential migration of human interneurons, as a consequence of an abnormality in neuronal progenitors.

RTT is the second most common cause of mental retardation in females, after Down syndrome. Few drugs have been shown to improve certain symptoms, whereas an effective cure is not achievable. Systematic studies of causative genetic mutations, molecular mechanisms of disease pathology, and targeted therapies are still urgently needed. Along with the applications of animal models, various brain organoid systems may serve as critical tools to facilitate these endeavors.

### Timothy syndrome (TS)

TS is a severe neurodevelopmental disease caused by genetic mutations in the gene CACNA1C encoding L-type calcium channel (LTCC) (Birey et al. 2017). Other genetic alterations have also been found associated with TS, including mutations in KCNQ1 (Wiener et al. 2008), KCNH2 (Sanguinetti et al. 1995), and SCN5A (Wang et al. 1996). It was reported that TS patient iPSC-derived cardiomyocytes exhibited consistent cellular defects (Yazawa et al. 2011). Cardiomyocyte-containing organoids thus could be one of the potential platforms for understanding TS mechanisms and identifying drug candidates (Yazawa et al. 2011).

In two similar studies, TS iPSC-derived neurons showed aberrant dendrite retraction, abnormal differentiation, and impaired activity-dependent gene expression (Krey et al. 2013; Simms and Zamponi 2014). The study also unveiled a mode through which CaV1.2 channels regulate RhoA signaling in the brain (Krey et al. 2013). Another study in the mouse model of TS demonstrated advanced maturation of oligodendrocyte progenitor cells (OPCs) and increased density of myelinating oligodendrocytes in the mouse brain (Cheli et al. 2018), implicating non-neuronal regulators in TS etiology.

Early investigation of LTCCs in animal models has indicated their critical functions in interneuron migration. However, it is challenging to investigate migratory behaviors of human interneurons in a dish, wherein in vivo physiological environments can be faithfully recapitulated. To solve this problem, human cortical spheroids (hCS) and human subpallium spheroids (hSS) were generated from iPSCs of TS patients. Patient-specific hCS and hSS were then assembled together to model tangential migration of human interneurons in 3D, with GABAergic interneurons labeled by Dlxi1/2b::eGFP. Live-cell tracking showed that human TS interneurons displayed an increase in saltation frequency but shorter saltation length compared to the control, resulting in a migratory deficiency (Birey et al. 2017). The migration defect of TS interneurons can be recovered by using LTCC blocker and cyclin-dependent kinase inhibitor, nimodipine and roscovitine, which reduce LTCCs activity (Cheli et al. 2018). More recently, it was found that phosphorylation of the myosin light chain (MLC) rescued the defect in the saltation length of TS interneurons during migration, and the abnormal saltation frequency could be restored by antagonism of GABA receptor (Birey et al. 2021).

Over the past few years, the use of iPSC-derived brain organoids, combined with calcium imaging, patch-clamp recording, and other approaches have revealed detailed mechanistic insights into ion channel function and dysfunction in TS, shedding light on potential treatment. More efforts are still desired to facilitate the translation of observations from laboratory models towards clinical practice.

### Tuberous sclerosis complex (TSC)

TSC is a developmental disorder affecting multiple organs, mainly including the skin, lungs, kidneys, and brain (Curatolo et al. 2008). Causative genetic mutations of TSC include alterations in tumor-suppressor genes TSC1 or TSC2, which form a complex with TBC1D7 to inhibit mTOR complex 1 (mTORC1), a regulator of cell proliferation and metabolism. Thus, TSC1/TSC2 mutant cells exhibit hyperactivation of effectors downstream mTOR pathway and increased cell proliferation (Li et al. 2017b; Lozovaya et al. 2014). TSC patients not only show neurological and psychiatric impairments, but also a high rate of epilepsy (Curatolo et al. 2015). Hallmark pathologies of TSC are cortical tubers containing a large number of astrocytes and dysmorphic neurons (Katz et al. 2017).

To establish human models of TSC, cortical spheroids were derived from hPSCs carrying either loss-of-function mutations in TSC1 or TSC2 (Blair et al. 2018). With homozygous, but not heterozygous, mutations in TSC1 and TSC2, cortical spheroids showed abnormal differentiation and hypertrophy of neurons and glia due to the failure in suppressing mTORC1 signaling (Blair et al. 2018). Notably, it was observed that an appropriate treatment window could be pivotal for effective reversal of developmental defects in TSC; in particular, early mTORC1 suppression is required to prevent neuronal differentiation defects, but mTORC1 hyperactivity may still re-emergence and affect differentiated cells if sustained mTORC1 inhibition is absent (Blair et al. 2018). Like neurogenesis, gliogenesis is critically involved in the development of TSC. It was observed in cortical organoids that TSC astrocytes showed increased proliferation and secreted more factors related to EGF signaling compared to the control. Such an abnormality could be an important regulator that causes an increase in inhibitory synapses, and consequently, alters the synaptic balance (Dooves et al. 2021). Thus, controlling gliogenesis also could be a potential strategy in TSC treatment.

### Down syndrome (DS)

DS is a complex genetic condition caused by triplication of chromosome 21 (T21), characterized by brain hypotrophy and intellectual disability. T21 brain has impaired neurogenesis, smaller brain hemispheres, and significantly reduced cerebellum (Chakrabarti et al. 2007; Golden and Hyman 1994; Wisniewski 1990). Many triplicated genes such as DYRK1A, amyloid precursor protein (APP), and OLIG1/2 have been verified as critical determinants of DS. For instance, mutations in DYRK1A cause less synaptic plasticity (Ahn et al. 2006), mild learning disability, but normal head circumference (Ronan et al. 2009). Triplication of the APP gene on human chromosome 21 is linked to Alzheimer’s disease (AD)-like neuropathology in DS patients (Prasher et al. 1998). Rodent models and 2D neural cultures have been essential in DS modeling. Nevertheless, in certain aspects, they may fall short in presenting DS-specific phenotypes. For instance, OLIG genes are vital for the production of GABAergic neurons, but the expression patterns of OLIG1 and OLIG2 in the embryonic ventral forebrain of human and mouse are different (Jakovcevski and Zecevic 2005; Watase and Zoghbi 2003).

Using human brain organoids derived from iPSCs of DS patients, it was found that DS ventral forebrain organoid overproduced OLIG2+ progenitors, and consequently showed an overproduction of inhibitory interneurons (GABAergic neurons), in accordance with the impaired excitatory and inhibitory balance in DS patients (Xu et al. 2019). In a 2D differentiation system, DS iPSC-derived interneurons showed a less complex morphology, altered subtype specification, and impaired migration capability (Huo et al. 2018).

Regarding dorsal forebrain development, cortical organoids were derived from iPSCs of T21 individuals (Tang et al. 2021). A significant decrease in neurogenesis was observed in DS samples, represented by diminished proliferation and decreased expression of layer II and IV markers. This could be causative to the smaller size of DS organoids. Importantly, suppression of the DSCAM/PAK1 pathway through multiple approaches was sufficient to restore deficits in neurogenesis and increase the size of DS organoids (Tang et al. 2021). Besides, multiple studies have indicated dosage-dependent effects of differentially-expressed genes on T21 among DS patients (Prandini et al. 2007). Currently, various methods including XIST silencing (Jiang et al. 2013), TKNEO-mediated silencing (Li et al. 2012), and CRISPR/Cas9-mediated editing (Ovchinnikov et al. 2018) have been utilized to correct target genes. It will be of importance to integrate these technologies into brain organoid modeling to investigate DS etiology and to explore potential therapeutics.

## Conclusions

**Fig. 2:**
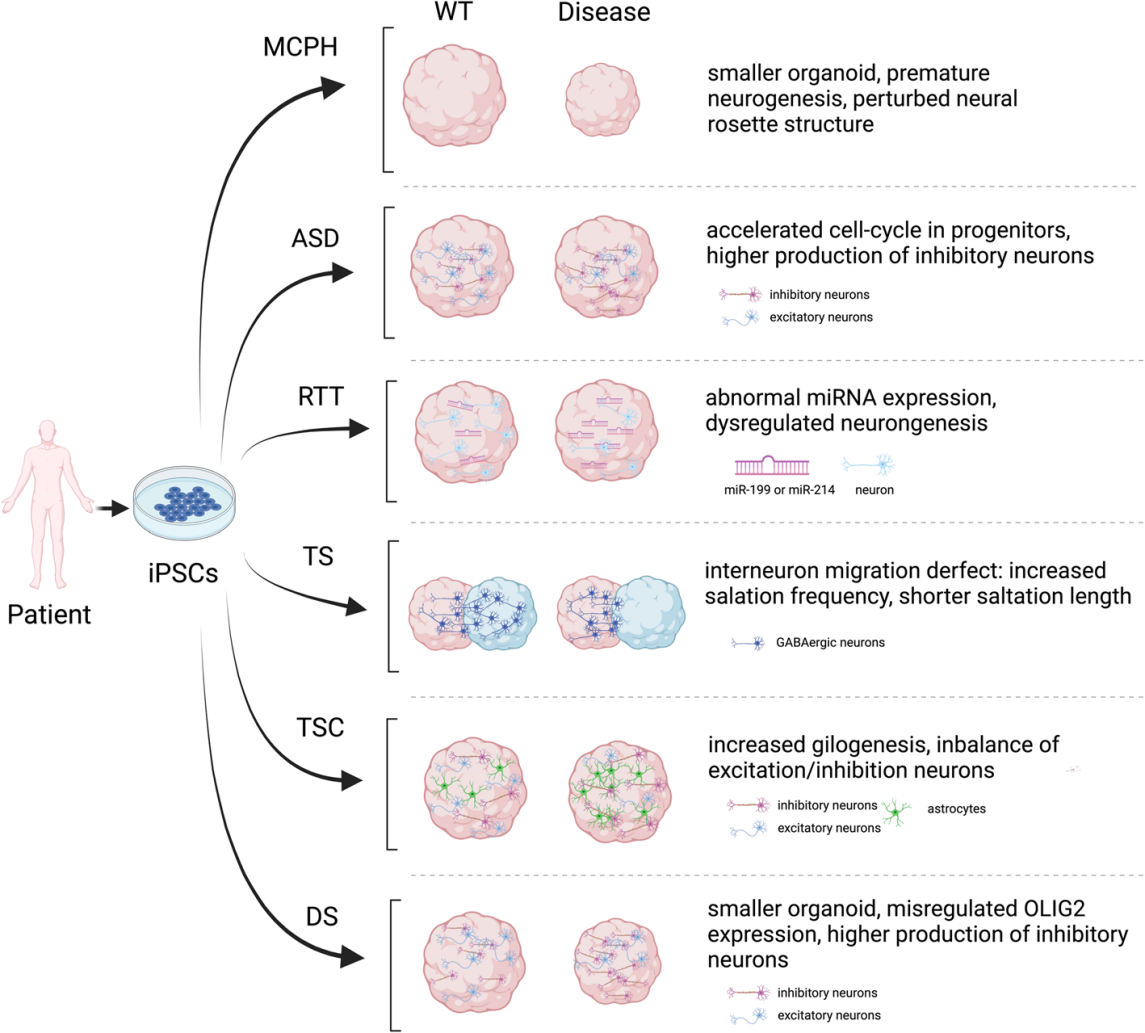
Brain organoid modeling of neurodevelopmental diseases. MCPH: Autosomal recessive primary microcephaly; ASD: Autism spectrum disorder; RTT: Rett syndrome; TS: Timothy syndrome; TSC: Tuberous sclerosis complex; DS: Down syndrome

Brain organoid models have been used to study a myriad of neurodevelopmental disorders that cause an increasing global burden, and valuable insights have been gained through the lens of these 3D in vitro models (Fig. 2; Table 1). Owing to their 3D microenvironment, their capacities of mimicking specific brain regions, and even reconstructing connections between distinct brain regions, brain organoids are important tools for modeling human neurodevelopment, exploring disease etiology, discovering complex cellular and molecular phenotypes, and identifying novel therapies.

**Table 1:** Human neurodevelopmental diseases modeled using brain organoids

Disease	Reference	Cell sources	Reprogramming or gene editing	Organoid types	Phenotypes	Relevant phenotypes in animal models
MCPH	Zhang et al. 2019	hESCs	CRISPR/Cas9-mediated WDR62 knockout	Cerebral organoids	Smaller organoids, premature differentiation of NPCs, reduction in oRGs proliferation	Mild effect on brain size, little change in neuroepithelium differentiation (Pulvers et al. 2010), reduced NPCs proliferation (Fish et al. 2006)
	Lancaster et al. 2013	Patient fibroblasts	Lentivirus expressing OSKM	Cerebral organoids	Premature neural differentiation, smaller organoids	
	Li et al. 2017a	Patient fibroblasts	Retrovirus expressing OSKM	Cortical organoids	Less organized neuroepithelium, fewer mature neurons, smaller organoids	
	Esk et al. 2020	hESCs	CRISPR/Cas9-mediated IER3IP1 knockout	Cerebral organoids	Smaller organoids, smaller neural rosettes	
ASD	Mariani et al. 2015	Patient fibroblasts	Retrovirus expressing OSKM	Telencephalic organoids	Increased production of GABAergic lineage (progenitors and neurons)	Decreased GABAergic neurotransmission (Wang et al. 2021), disruption of cortico-striatal synapses (Peça et al. 2011)
	Schafer et al. 2019	Patient fibroblasts	Retrovirus expressing OSKM	Forebrain organoids	Abnormal morphology of cortical neurons	
	Wang et al. 2017	Patient fibroblasts	CRISPR/Cas9-mediated CHD8 knockout	Cerebral organoids	Increased production of GABAergic interneurons	
RTT	Mellios et al. 2018	Patient fibroblasts	Retrovirus expressing OSKM	Cerebral organoids	Dysregulation in miRNA expression and neurogenesis	Abnormal morphology of cortical neurons (Jugloff et al. 2005), alteration in interneuron subtypes (Tomassy et al. 2014)
	Xiang et al. 2020	hESCs	TALEN- and CRISPR/Cas9-mediated MECP2 knockout and knockin	hMGEOs and hCOs	Abnormal morphology and function of GABAergic interneurons	
	Gomes et al. 2020	Patient fibroblasts	Retrovirus expressing OSKM	Dorsal forebrain organoids, ventral forebrain organoids, assembloids	Premature neurons with functional defect	
TS	Krey et al. 2013	Patient fibroblasts	Modified mRNA cocktail of OSKM	Cerebral organoids	Aberrant dendrite retraction and dyregulation of RhoA signaling in cortical neurons	Disrupted neurite outgrowth and impaired migration of cortical neurons (Kamijo et al. 2018; Panagiotakos et al. 2019)
	Birey et al. 2017	Patient fibroblasts	Retrovirus expressing OSKM	hCS, hSS, assembloids	Abnormal interneuron migration	
TSC	Blair et al. 2018	hESCs	CRISPR/Cas9-mediated TSC1 and TSC2 knockout	Cortical organoids	Biased toward astroglial differentiation, activation of mTORC1 signaling	Increased astroglial differentiation (Uhlmann et al. 2002), decreased glutamate transport (Zeng et al. 2010)
	Dooves et al. 2021	Patient fibroblasts	Lentivirus expressing OSKM	Cortical organoids	Increased gilogenesis, increased GABAergic synapses	
DS	Xu et al. 2019	Patient fibroblasts	Sendai virus expressing OSKM	Forebrain organoids	Overproduction of GABAergic interneurons	Increased GABAergic neurotransmission (Kleschevnikov et al. 2012; Martínez-Cué et al. 2013), smaller brain volume (Belichenko et al. 2009)
	Tang et al. 2021	Patient fibroblasts	Sendai virus expressing OSKM	Cerebral organoids	Smaller organoids, dysregulation of the DSCAM/PAK1 pathway	

Cell self-organization is an essential basis for organogenesis (Lancaster and Knoblich 2014). However, cell self-organization also brings about unpredictability and variability to hPSC-derived organoid models. Several other challenges also exist for current brain organoid technology, which hinders brain organoids from fully recapitulating many key aspects of the human brain. These include the lack of functional vasculatures, incomplete cellular compositions, preliminary structural organizations, and functional neuronal networks, etc. Thus, more efforts are required to build improved brain organoid methodologies to widen the applicability of brain organoids. In addition, gene-editing tools, especially CRISPR/Cas9-based technologies, have been widely applied together with brain organoid technologies. It is conceivable that the incorporation of more interdisciplinary technologies will further enable the application of brain organoids.

Although brain organoids have been successfully applied to model various diseases in a dish, multiple challenges still exist. As a starting point, ideally, a larger pool of iPSC lines, from both healthy donors and patients, is always desired to obtain a more reliable conclusion. Especially given the potential variability among brain organoids samples, even for those derived from the same donor, the scale of samples for comparison is always critical when deciphering disease-related phenotypes. This is particularly critical when studying diseases with complex genetic alterations. Thus, on one hand, the brain organoid systems remain to be optimized to decrease sample variation. On the other hand, more iPSC lines, or gene-edited ESC lines, need to be established, which however could be costly and not always achievable.

Multiple lines of effort have been taken to understand and control sample variations during brain organoid generation. It should be noted that variations are also highly dependent on specific differentiation protocols. By using single-cell RNA-seq, it was demonstrated that with defined guidance of differentiation, brain organoids showed relatively limited variations, as revealed by similar cell-type composition and proportions among different samples (Velasco et al. 2019; Yoon et al. 2019). Nevertheless, since the culture of brain organoids generally takes months, to avoid potential variations during this process, careful quality controls should be considered. These include, but not limited to, the validation of hPSC quality, consistency among initial EBs, consistency among medium and supplements (e.g., the compounded Matrigel solution), similar culture environments (e.g., incubators and bioreactors), etc. Besides, to facilitate cell self-organization, various studies have applied engineering approaches for guided morphogenesis (Hofer and Lutolf 2021). In particular, extracellular matrix (ECM) is critical for cell-cell communication and organogenesis (Loganathan et al. 2016), whereas high variations may exist among Matrigel batches. Thus, one essential task is to design more controllable and reproducible ECM for organoid culture. To address this challenge, synthetic hydrogel ECM by design has been shown to fit key environmental features for organoid development (Below et al. 2021) and could be a valuable strategy to consider in future studies.

When interpreting observations from in vitro models, whether and how phenotypes revealed in brain organoids (or 2D cell cultures) correlate with symptoms in patients are essential questions to be addressed as well. Since animal models also provide irreplaceable insights in many cases, careful interpretations of observations from brain organoids are required, particularly when inconsistent phenotypes are observed in different model systems (Table 1). Further, neurodevelopmental diseases have a wide range of occurring time, ranging from embryonic development to early childhood. Thus, to which extent should the brain organoids be cultured and monitored is an important aspect to consider, which may vary from disease to disease. Particularly, for late-onset diseases, long-term culture of brain organoids may be required to have better manifestations of disease-relevant alterations. This, however, again requires optimizations of the brain organoid system to safeguard the healthy development of brain organoids during long-term culture.

As a novel in vitro platform to recapitulate normal and abnormal development of the human brain, together with other model systems, brain organoids hold the promise to advance future studies in disease modeling, drug discovery, and regenerative medicine. Nevertheless, as a newly-emerged technology, further efforts are also required to build better organoid models of the human brain, and to explore more faithful presentations of human diseases with such models.

## Data Availability

Not applicable.

## Abbreviations

hPSCs: Human pluripotent stem cells
iPSCs: Induced pluripotent stem cells
ESCs: Embryonic stem cells
PSCs: Pluripotent stem cells
3D: Three-dimensional
MCPH: Autosomal recessive primary microcephaly
SZ: Schizophrenia
ASD: Autism spectrum disorder
RTT: Rett syndrome
TS: Timothy syndrome
TSC: Tuberous sclerosis complex
DS: Down syndrome
EBs: Embryonic bodies
NPCs: Neural progenitor cells
CSF: Cerebrospinal fluid
ECs: Endothelial cells
HUVECs: Human umbilical vein endothelial cells
vRG: Ventral radial glial
oRG: Outer radial glial
INM: Interkinetic nuclear migration
ER: Endoplasmic reticulum
ERAD: ER-associated protein degradation
MGE: Medial ganglionic eminence
hMGEOs: Human medial ganglionic eminence (MGE) organoids
hCOs: Human cortical organoids
LTCC: L-type calcium channel
OPCs: Oligodendrocyte progenitor cells
hCS: Human cortical spheroids
hSS: Human subpallium spheroids
mTORC1: mTOR complex 1
T21: Triplication of chromosome 21
ECM: Extracellular matrix
